# Effect of *Salicornia herbacea* on Osteoblastogenesis and Adipogenesis *in Vitro*

**DOI:** 10.3390/md12105132

**Published:** 2014-10-10

**Authors:** Fatih Karadeniz, Jung-Ae Kim, Byul-Nim Ahn, Myeong Sook Kwon, Chang-Suk Kong

**Affiliations:** 1 Marine Biotechnology Center for Pharmaceuticals and Foods, Silla University, Busan 617-736, Korea; E-Mails: f_karadeniz@yahoo.com (F.K.); jale8469@gmail.com (J.-A.K.); 2 Department of Food and Nutrition, College of Medical and Life Science, Silla University, Busan 617-736, Korea; E-Mail: 1506audtnr@hanmail.net; 3 Department of Organic Material Science and Engineering, Pusan National University, Busan 609-735, Korea; E-Mail: icetwig@naver.com

**Keywords:** *Salicornia herbacea*, osteoporosis, adipogenesis, osteoblastogenesis

## Abstract

Bone-related complications are among the highest concerning metabolic diseases in the modern world. Bone fragility and susceptibility to fracture increase with age and diseases like osteoporosis. Elevated adipogenesis in bone results in osteoporosis and loss of bone mass when coupled with lack of osteoblastogenesis. In this study the potential effect of *Salicornia herbacea* extract against osteoporotic conditions was evaluated. Adipogenesis inhibitory effect of *S.* *herbacea* has been evidenced by decreased lipid accumulation of differentiating cells and expression levels of crucial adipogenesis markers in 3T3-L1 pre-adipocytes. *S.* *herbacea* treatment reduced the lipid accumulation by 25% of the control. In addition, mRNA expression of peroxisome proliferator-activated receptor (PPAR)γ, CCAAT/enhancer-binding protein (C/EBP)α and sterol regulatory element binding protein (SREBP)1c were inhibited by the presence of *S. herbacea*. Bone formation enhancement effect of *S.* *herbacea* was also confirmed in MC3T3-E1 pre-osteoblasts*.* The presence of *S. herbacea* significantly elevated the alkaline phosphatase (ALP) activity by 120% at a concentration of 100 μg/mL in differentiating osteoblasts. *S. herbacea* also significantly increased the expression of osteoblastogenesis indicators, ALP, bone morphogenetic protein (BMP)-2, osteocalcin and collagen type I (collagen-I). In conclusion, *S. herbacea* possess potential to be utilized as a source of anti-osteoporotic agent that can inhibit adipogenesis while promoting osteoblastogenesis.

## 1. Introduction

Natural sources that can be used for the treatment or prevention of diseases have been of much interest during the past decades. In this context, extracts and compounds from plants are among the main targets of studies [1,2]. Several studies in the past reported various biologically active plants with marine plants allocating a large part [3]. Due to the harsh conditions of marine environments and the need for strong protection, marine organisms produce unique substances. Plant extracts and compounds have been intensely studied in order to fully understand their action mechanism against common complications such as oxidative stress and inflammation [4,5,6], as well as diseases with high morbidity and mortality rates including diabetes, cancer, obesity and AIDS [7,8,9]. The halophyte *Salicornia herbacea* is endemic to the western coast of the Korean peninsula and a part of folk medicine due to its effect on constipation, diabetes, obesity, *etc.*, [10]. As expected, a number of experiments have credited *S. herbacea* for various bioactivities including antioxidative, antiinflamatory, antihyperglycemic and antihyperlidemic [11,12,13]. It was proposed that *S. herbacea* contained active flavonoids [14,15,16]. However, very few investigations have been carried out in order to evaluate the effect of *S. herbacea* on osteoporosis.

Bone-related diseases, namely osteoporosis and age-related osteopenia are reported to be associated with bone mass loss due to lack of osteoblastogenesis [17]. Studies showed that glucocorticoid treatment also caused an increase in bone adipocytes, which finally resulted in fractures and osteoporosis [18]. Several mechanisms have been suggested for possible cause of bone mass imbalance. Peroxisome proliferator activated receptor (PPAR)γ is confirmed to play crucial roles in the outcome of bone marrow mesenchymal cell differentiation [19]. Both *in vitro* and *in vivo* mechanism studies demonstrated that the activation of PPARγ promotes adipogenesis. Likewise, suppression of PPARγ pathway was shown to inhibit adipogenesis and stimulate osteoblast differentiation depending on the binding ligand [20]. Several diabetic drugs as ligands of PPARγ activate adipogenesis and lower blood glucose. However, this activation also causes problems in bone mass by favoring adipogenesis of bone mesenchymal cells and deteriorating the bone mass balance. Diabetic drugs, obesity-related factors and long chain fatty acids were confirmed to be activating ligands for PPARγ [21]. Overall, in this study *S. herbacea* was tested for its potential effect on adipogenesis of pre-adipocytes and pre-osteoblast differentiation with a possible intervention in PPARγ pathway.

## 2. Results

### 2.1. Effect of S. herbacea on Differentiation of 3T3-L1 Pre-Adipocytes

**Figure 1:**
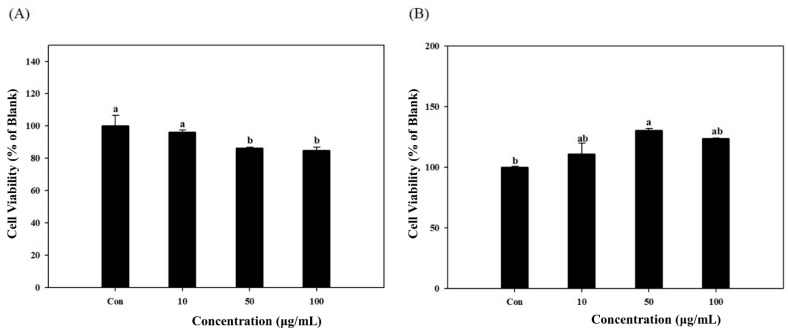
Cytotoxicity levels of *S.* *herbacea* on the viability of 3T3-L1 (**A**) and MC3T3-E1 (**B**) cells. Cell viability was observed by MTT assay and presented as percentage value of untreated undifferentiated blank cells. Values are means ± SD (*n* = 3). ^a,^^b ^Means with different letters are significantly different (*p* < 0.05) by Duncan’s multiple range test. (Con: Differentiated untreated cells).

Prior to assays, the cytotoxicity of samples was tested *in vitro* using the 3T3-L1 and MC3T3-E1 cells which were used in further experiments. The cytotoxic effect of *S. herbacea* extracts in 3T3-L1 (Figure 1A) and MC3T3-E1 (Figure 1B) cells are presented. At a concentration of 10 μg/mL, there was no cytotoxicity for both cell lines while the two highest concentrations (50 and 100 μg/mL) caused slight decrease in cell viability of 3T3-L1 cells, indicating a small effect on cell viability at higher concentrations. However, no cytotoxic effect was observed on MC3T3-E1 cells at concentrations of 50 and 100 μg/mL.

**Figure 2:**
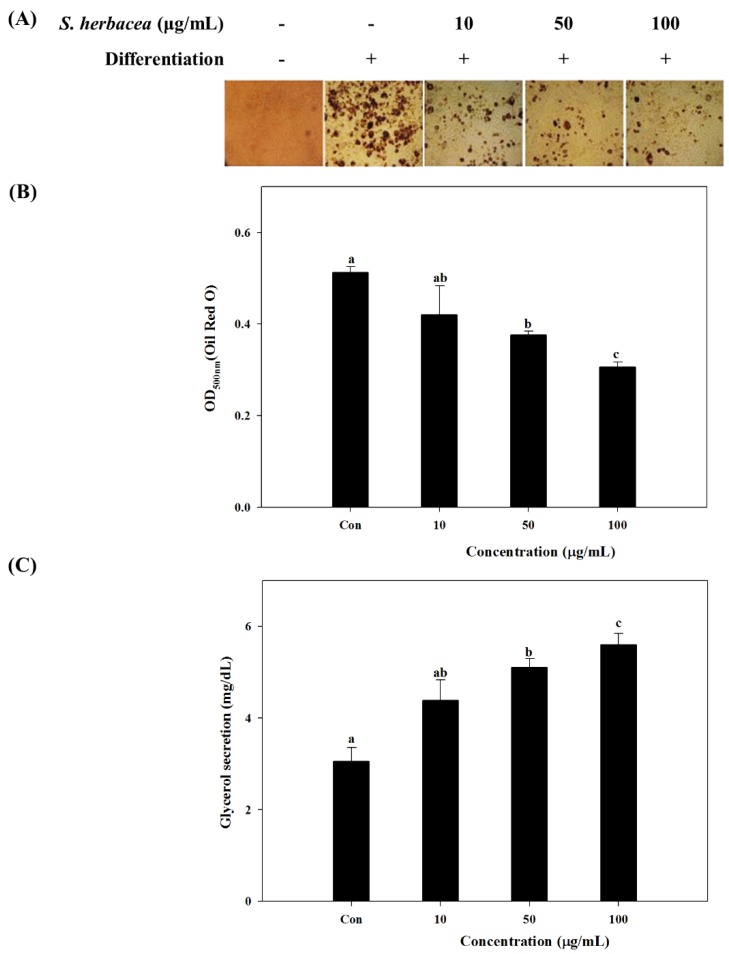
Effect of *S.* *herbacea* on adipogenic differentiation as stained cell images following Oil Red O staining of intracellular lipids (**A**), quantification of lipid accumulation by the absorbance of eluted intracellular stain (**B**) and glycerol secretion into culture medium during final differentiation (**C**) of mature 3T3-L1 adipocytes. Values are means ± SD (*n* = 6). ^a–^^c^ Means with different letters are significantly different (*p* < 0.05) by Duncan’s multiple range test.

In order to evaluate the effect of *S. herbacea* on adipogenesis, 3T3-L1 mouse pre-adipocytes were differentiated into mature adipocytes in the presence and absence of *S. herbacea* extract. Lipid accumulation which is a key indicator of successful maturation into adipocytes was used to analyze the anti-adipogenesis effect. Lipid accumulation is observed by staining of intracellular triglycerides with Oil-Red O. Cell images after staining indicated that the presence of *S. herbacea* decreased the intracellular lipid amount (as red colored areas in cell images) in a dose-dependent manner (Figure 2A). Observable lipid accumulation stained by Oil-Red O was at a minimum compared to all other cases, in the case of the highest concentration treated (100 μg/mL). Inhibited lipid accumulation was quantified by the elution of accumulated Oil Red O stain. Absorbance values of intracellular accumulated stain are shown in Figure 2B. Quantification of lipid accumulation was in accordance with the cell images. The presence of *S. herbacea* lowered the intracellular stain amount as an indicator of suppressed lipid accumulation. In order to assess the mechanism behind the lowering of lipid amounts, glycerol release into the culture medium was quantified. Excessive glycerol secretion of adipocytes is a key marker for triglyceride lipolysis. In the presence of *S. herbacea*, glycerol secretion of differentiated cells increased from 3.09 ± 0.51 to 4.64 ± 0.62, 5.26 ± 0.40 and 5.79 ± 0.32 at concentrations of 10, 50 and 100 μg/mL, respectively (Figure 2C).

**Figure 3:**
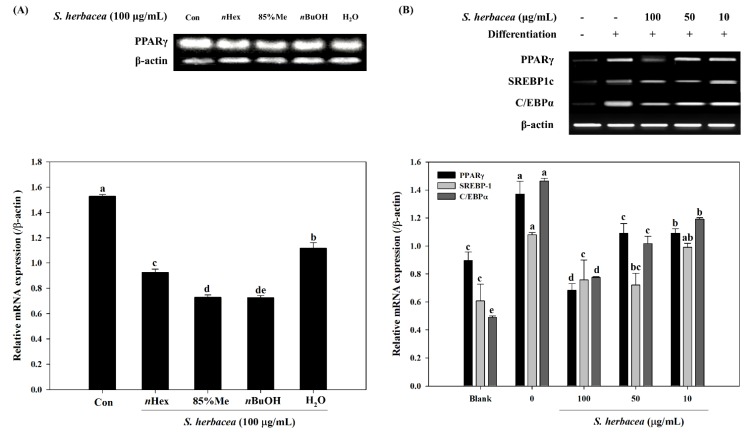
Effect of *S.* *herbacea* extract fractions (**A**) and the crude extract (**B**) on the mRNA expression levels of adipogenic target genes in 3T3-L1 adipocytes assessed by RT-PCR and band quantification. Values are means ± SD (*n* = 3). ^a–^^e^ Means with different letters are significantly different (*p* < 0.05) by Duncan’s multiple range test.

Next, in order to evaluate whether *S. herbacea* affects the expression of key transcription factors for adipogenesis, RT-PCR experiments were carried out. Possible changes in the mRNA expressions of PPARγ, differentiation-dependent factor 1/sterol regulatory element-binding protein (SREBP1c) and CCAAT/enhancer-binding proteins (C/EBPα) were observed. Different solvent fractions were tested for their ability to suppress PPARγ mRNA expression. Among four tested fractions (*n*Hexane, 85% MeOH, *n*BuOH and H_2_O), the *n*BuOH fraction decreased the PPARγ expression most compared to control (Figure 3A). In addition, treatment significantly suppressed the expression of PPARγ, SREBP1c and C/EBPa, compared to fully differentiated control adipocytes (Figure 3B). Hence, *n*BuOH was considered for further activity based-isolation of compounds. Other fractions also showed an observable decrease in mRNA expression, the H_2_O extract being the least effective.

### 2.2. Effect of S. herbacea on Osteogenic Differentiation of MC3T3-E1 Pre-Osteoblasts

**Figure 4:**
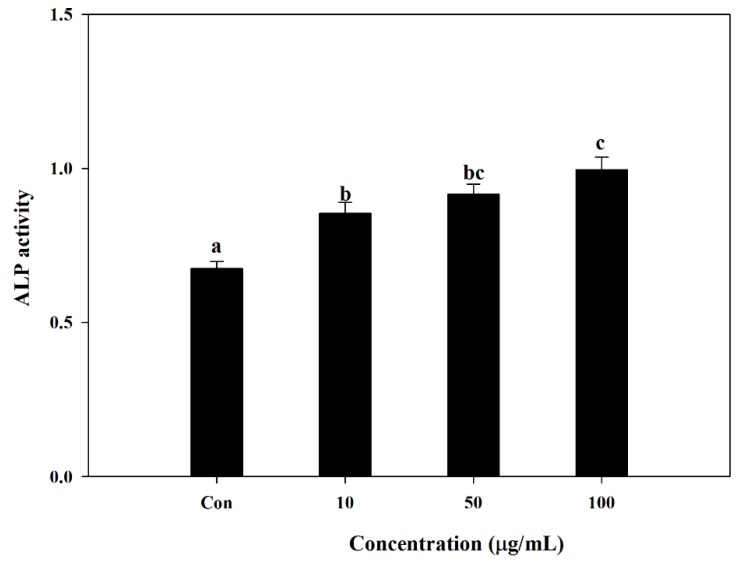
Effect of *S.* *herbacea* on alkaline phosphatase (ALP) activity in MC3T3-E1 cells after 14 days of differentiation. Values are means ± SD (*n* = 6). ^a–^^c^ Means with different letters are significantly different (*p* < 0.05) by Duncan’s multiple range test.

*S. herbacea* was tested for its ability to enhance osteoblast differentiation in mouse MC3T3-E1 pre-osteoblasts. Cells were treated with different concentrations of *S. herbacea* during the differentiation process. Measurement of alkaline phosphatase (ALP) activity of MC3T3-E1 osteoblasts was used as a marker for osteoblastogenesis. Effect of *S. herbacea* on ALP activity was measured by a commercial kit according to the manufacturer’s protocol. Results suggested an enhancing ability of *S. herbacea* towards ALP-indicated osteoblastogenesis. Compared to differentiated control cells, *S. herbacea* treatment enhanced the ALP activity by increasing the absorbance values of reaction mixtures from 0.681 ± 0.092 to 0.814 ± 0.081, 0.896 ± 0.094 and 1.049 ± 0.102 at concentrations of 10, 50 and 100 μg/mL, respectively (Figure 4).

**Figure 5:**
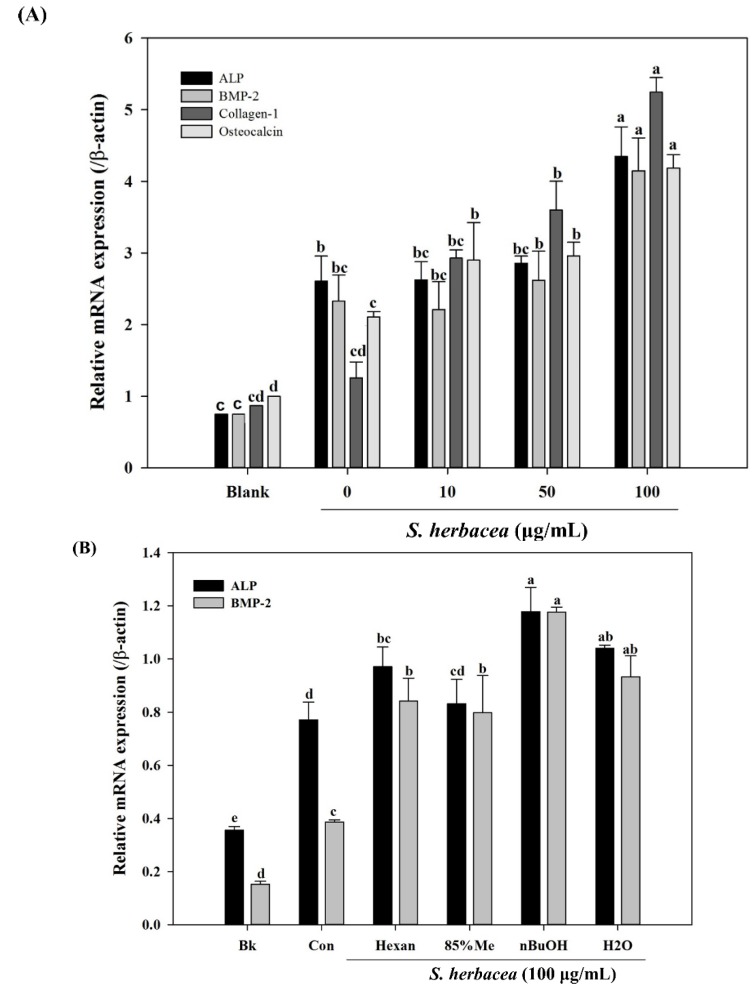
Effect of *S. herbacea* extract (**A**) and fractions (**B**) on mRNA expression of key osteblastogenesis markers in MC3T3-E1 osteoblasts according to real time PCR assay. Values are means ± SD (*n* = 3). ^a–e^ Means with different letters are significantly different (*p* < 0.05) by Duncan’s multiple range test.

**Figure 6:**
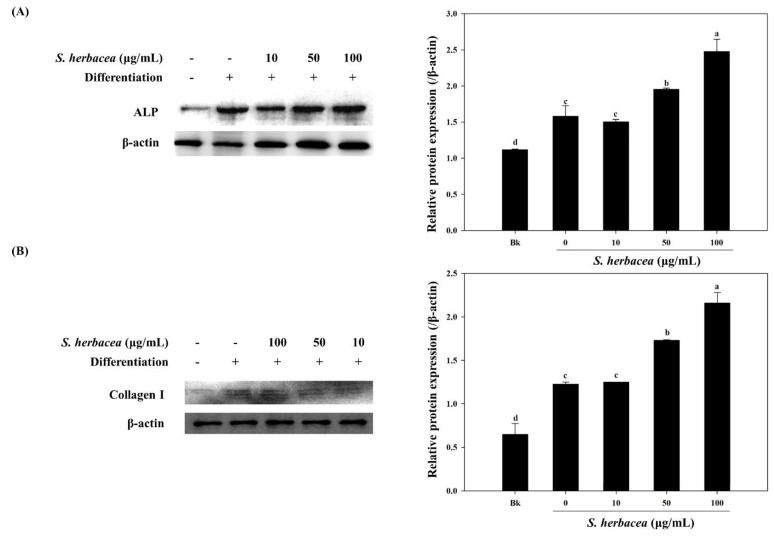
Effect of *S. herbacea* extract on protein levels of ALP (**A**) and collagen (**B**) in differentiated MC3T3-E1 osteoblasts. Values are means ± SD (*n* = 3). ^a–d^ Means with different letters are significantly different (*p* < 0.05) by Duncan’s multiple range test.

During osteogenic differentiation, MC3T3-E1 pre-osteoblasts were treated with *S. herbacea* extract at concentrations of 10, 50 and 100 μg/mL. In order to detect and evaluate the differentiation of pre-osteoblasts, following incubation during 14 days of differentiation, gene expression of key osteogenic markers, namely ALP, BMP-2, osteocalcin, collagen-I, and intracellular levels of ALP and collagen-I proteins were quantified by real-time PCR and Western blotting, respectively. *S. herbacea* enhanced the expression of ALP, BMP-2, osteocalcin and collagen-I mRNA in a dose-dependent manner (Figure 5A). Fractions of extract were shown to enhance the mRNA expression of osteogenesis markers in a similar way to suppressing adipogenesis indicators (Figure 5B). As expected, treatment with *S. herbacea* notably increased intracellular levels of ALP and collagen-I proteins in a similar fashion to mRNA levels (Figure 6). According to the results, the presence of *S. herbacea* enhanced the expression of osteogenic marker proteins in MC3T3-E1 cells.

**Figure 7:**
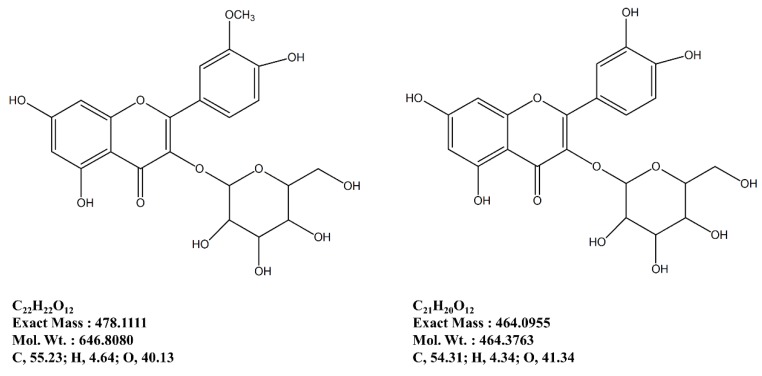
Isolated flavonoid glycosides as anti-osteoporotic ingredients of *S.* *herbacea*.

Following a bioactivity-directed isolation method, two formerly known flavonoid glycosides were isolated from the *n*BuOH fraction of *S. herbacea* as a part of ongoing research (Figure 7). In total accordance with our earlier report, the isolated flavonoid glycosides were confirmed by comparison of basic structural data (data not shown) [15].

## 3. Discussion

The main age-related metabolic diseases are the major causes of diminished life quality for the elderly. Additionally, recent studies indicate an increasing trend for the younger generation to be diagnosed with obesity, type-2 diabetes and osteoporosis [16,22,23]. Scientists are turning their attention to natural products to use to intervene with the progression of the aforementioned diseases due to bioavailability, biodegradation and fewer side effects. In this context, marine-based organisms are of high interest to pharmaceutical researchers as most marine plants and animals live in extreme conditions, which results in the need for unique compounds for survival. By means of this promising situation, past decades have been fruitful in ways of natural bioactive substance development. Numerous marine plants have been studied intensively in order to discover bioactive chemicals that can act against cancer, tumor growth, oxidative stress, diabetes, obesity and osteoporosis [5,24,25]. Halophytes are also plants that survive under extreme conditions such as high concentrations of salt and harsh climate changes. In this context, the halophyte *S. herbacea* was examined here for possible compounds with health beneficiary effects. Reports had already stated that *S. herbacea* contains flavonoid glycosides and polysaccharides, which are natural antioxidants [15].

In this study, we examined the ability of *S. herbacea* to relieve osteoporosis conditions with a regulatory mechanism towards adipogenesis/osteoblastogenesis imbalance. Uncontrolled inducement of adipogenesis was shown to create a bone mass imbalance which resulted in elevated fragility and susceptibility. Differentiation of both pre-adipocyte and mesenchymal cells is partly regulated by PPARγ signaling [26]. Obesity conditions were recently linked to increased osteoporosis progression in proportional to elevated adipokines [27]. Adipokines and triglycerides trigger in a similar way an increase in adipogenesis, which is required to be in balance with osteoblastogenesis for healthy bones. Regulation of this imbalance is crucial to prevent and treat osteoporosis, especially when developed under obesity.

Our results showed a strong inhibition on lipid accumulation of differentiated 3T3-L1 adipocytes by *S. herbacea* treatment. Glycerol assay results showed that cells treated with *S. herbacea* released more glycerol to the culture medium. Furthermore, a possible mechanism behind the anti-adipogenic effect of *S. herbacea* was evaluated by assessing expression levels of adipogenesis regulator factors, PPARγ, SREBP1c and C/EBPα. In addition to lowering lipid accumulation, *S. herbacea* also lowered the expression of the aforementioned adipogenesis factors. Results indicated that *S. herbacea* inhibited not only lipid accumulation, but also adipogenesis. In order to be used for further bioactivity-directed isolation, comparison of *S. herbacea* solvent fractions was carried out. Among all fractions, *n*BuOH was found to be the most active to inhibit PPARγ mRNA expression. In addition, Kong *et al.* [14] also reported that two glycosides isolated from *S. herbacea* were shown to possess chemoprotective effect against cancer through matrix metalloproteinase inhibition.

Increase in bone adipocytes is accompanied by severe fragility that defines osteoporosis. In order to relieve the deteriorated stem cell differentiation, inducing differentiating cells towards osteoblastogenesis is considered to be a crucial treatment step. Our results showed that *S. herbacea* inhibited adipogenesis by possible interaction with the PPAR-γ pathway and lipolysis. Results also indicated that *S. herbacea* might attenuate the imbalance of bone cell differentiation towards osteoblastogenesis.

In osteoporosis, mesenchymal cell perseverance towards adipogenesis is also accompanied by diminished osteoblastogenesis [28,29]. If an increase in adipogenesis is coupled with a decrease in osteoblastogenesis, bone loses its sturdiness and tends to be more fragile.

We examined the ability of *S. herbacea* extract to enhance osteoblast differentiation in MC3T3-E1 pre-osteoblasts. Differentiating MC3T3-E1 pre-osteoblasts were introduced to *S. herbacea* in different concentrations. Following a full differentiation, alkaline phosphatase (ALP) activity of cell lysates was evaluated. The alkaline phosphatase level is known to be an indicator of successful osteoblast differentiation, as it plays a crucial role in the mineralization of bone [30]. In this regard, results indicated that the presence of *S. herbacea* elevated the ALP activity suggesting a role in the mineralization of osteoblasts at all treated concentrations, indicating an enhancement towards bone formation.

Bone formation is carried out by a distinctive and well-studied pathway of factors and proteins. In this cascade of signaling, bone morphogenetic protein (BMP) 2, 4 and 7 and osteocalcin are some of the key factors elevated at gene expression levels [31]. During osteoblastogenesis, osteocalcin has also been reported to be a cell marker for the final differentiation state. On the other hand, BMPs are known to enhance expression of alkaline phosphatase (ALP), type I collagen (collagen-I) and other non-collagenous bone proteins as indicators for successful osteoblastogenesis [30]. Therefore, mRNA expression of ALP, BMP-2, osteocalcin and collagen-I were assessed in the absence and presence of *S. herbacea*. After full maturation into osteoblasts, RT-PCR experiments suggested an increasing trend towards expression of the aforementioned markers in cells treated with *S. herbacea*, a result confirmed for ALP and collagen-I by western blot. In the light of these results, it was suggested that *S. herbacea* enhanced osteoblast differentiation while inhibiting adipogenesis in pre-adipocytes. Ha *et al.* [32] also reported that *S. herbacea* might be a potential source of antioxidant agents because of its effect on ovariectomy-induced oxidative stress, which is considered to cause age-related diseases including osteoporosis. Taken together, our results on *S. herbacea* extracts provide evidence for an inhibitory effect on adipogenesis while enhancing osteoblast differentiation. Therefore, *S. herbacea* is proposed as a promising source of bioactive agents for the effective prevention and treatment of osteoporosis.

In this regard, following an earlier reported isolation method adapted for anti-osteoporosis bioactivity-directed flow, two formerly known flavonoid glycosides were isolated [14]. These glycosides, namely isorhamnetin 3-*O*-β-d-glucoside and quercetin 3-*O*-β-d-glucoside are suggested to be possible bioactive reagents of *S. herbacea*, responsible for relieving the effect of osteoporosis through regulation of adipogenesis/osteoblastogenesis imbalance by inhibiting PPARγ pathway and enhancing bone formation. Isolation of glycosides from the *n*BuOH fraction was expected as this fraction was the most active for inhibiting adipogenesis and enhancing osteoblastogenesis. Several other flavonoid glycosides were isolated and evaluated for their anti-adipogenic activities. Studies also showed the possible absorption and bioactivity mechanisms of dietary flavonoids through the small intestine. Our previous results [14,15] were also in accordance with current assays and suggested that isorhamnetin 3-*O*-β-d-glucoside and quercetin 3-*O*-β-d-glucoside are strong bioactive substituents of *S. herbacea*. On the other hand, isorhamnetin 3-*O*-β-d-glucoside and quercetin 3-*O*-β-d-glucoside were also shown to act on inhibition of differentiation of 3T3-L1 cells through AMPK/MAPK pathways. Notoya *et al.* [33] suggested an inhibitory effect of the flavonoid, quercetin, on proliferation, differentiation and mineralization of osteoblasts. However, Kim *et al.* [34] also stated that quercetin was able to inhibit proliferation while elevating the osteogenic differentiation of adipose stromal cells. In such a case, quercetin derivatives might also show the same distinct bioactivity on adipogenesis and osteoblastogenesis as our preliminary results indicated. The isolated derivative of quercetin, quercetin 3-*O*-β-d-glucoside has the quercetin backbone with a glucoside side-chain containing -OH branches similar to phloroglucinol derivatives. Considering that phloroglucinol derivatives have been reported to have anti-adipogenesis and pro-osteoblastogenesis activities, it could be suggested that these flavonoids might be responsible for the reported bioactivity of S. herbacea. Nonetheless, the *n*BuOH fraction of *S. herbacea* showed strong effects on adipogenesis and osteoblastogenesis and two flavonoid glycosides were isolated from the fraction. However, further studies to reveal the real action mechanisms of isolated compounds and their efficiency on adipogenic and osteogenic differentiation are needed. According to the results, the *n*BuOH fraction of *S. herbacea* might contain other bioactive constituents that are responsible for anti-adipogenic and pro-osteoblastogenic effects. In the future, coupled with *in vivo* assays, results of this study will help to evaluate the true potential of *S. herbacea*, as a potential source of compounds against obesity-related osteoporosis.

## 4. Experimental Section

### 4.1. Plant Materials

The whole plant of *S. herbacea* was briefly dried under shade and kept at −25 °C until use. The air-dried sample of *S. herbacea* was chopped into small pieces and extracted for 24 h with CH_2_Cl_2_ (3 L × 2) at room temperature. After removal of the solvent, the residue was re-extracted for 24 h with MeOH (3 L × 2) at room temperature.

The combined crude extracts (50 g) were suspended between CH_2_Cl_2_ and water. The organic layer was further partitioned between 85% aqueous MeOH and* n*-hexane and then the aqueous layer was fractioned with *n*-BuOH and H_2_O, respectively, to afford the *n*-hexane (3.2 g), 85% aq. MeOH (12.1 g), *n*-BuOH (16.3 g) and water (15.5 g) fractions.

### 4.2. Cell Culture and Adipocyte/Osteoblast Differentiations

Murine 3T3-L1 pre-adipocytes were seeded in 6-well plates at a density of 2 × 10^5^ cells/well prior to experiments and grown to confluence in Dulbecco’s modified Eagle’s medium (DMEM) with 10% fetal bovine serum (FBS) at 37 °C in a humidified atmosphere of 5% CO_2_. At 1 day postconfluence (designated “day 0”), a mixture of 3-isobutyl-1-methylxanthine (0.5 mM), dexamethasone (0.25 M) and insulin (5 µg/mL) in DMEM containing 10% FBS was introduced into cells in order to induce cell differentiation. After 48 h (day 2), DMEM containing 10% FBS supplemented with insulin (5 µg/mL) was introduced to cells following removal of induction medium. While replacing this medium with a fresh one every two days, *S.* *herbacea* extract was administered to the culture medium from day 0 to day 6 for Oil Red O and RT-PCR experiments and day 6 to day 8 for the glycerol secretion assay.

Murine osteoblast-like MC3T3-E1 cells were seeded in 6-well plates at a density of 1 × 10^5^ cells/well and grown to confluence in α-Modified minimal essential medium (αMEM) supplemented with 10% heat-inactivated fetal bovine serum (FBS), 1 mM sodium pyruvate, 100 units/L penicillin and 100 mg/L streptomycin at 37 °C in a humidified atmosphere of 5% CO_2_. The cells were induced into osteoblastogenesis by adding of ascorbic acid and β-glycerophosphate into medium for five days under the conditions of the earlier report [35]. Following confluence, the cell differentiation was initiated with culture medium containing 50 μg/mL ascorbic acid and 10 mM β-glycerophosphate for three days. Then, the induction medium was removed and the cell monolayer was washed twice with phosphate buffered saline (PBS). *S.* *herbacea* extract was administered to the culture medium prior to further incubation of 48 h.

### 4.3. Cytotoxicity Determination Using MTT Assay

Cytotoxic levels of the *S. herbacea* on cultured cells were measured using MTT (3-(4,5-dimethylthiazol-2-yl)-2,5-diphenyltetrazolium bromide) assay, which is based on the conversion of MTT to MTT-formazan by mitochondrial enzyme. The cells were grown in 96-well plates at a density of 5 × 10^3^ cells/well. After 24 h, the cells were washed with fresh medium and were treated with control medium or the medium supplemented with *S. herbacea*. After incubation for seven days while changing the medium and re-treating the samples every two days, cells were rewashed and 100 μL of MTT solution (1 mg/mL) was added and incubated for 4 h. Finally, 100 μL of DMSO was added to solubilize the formed formazan crystals and the amount of formazan crystal was determined by measuring the absorbance at 540 nm using a GENios^®^ microplate reader (Tecan Austria GmbH, Grödig, Austria). Relative cell viability was determined by the amount of MTT converted into formazan crystal. Viability of cells was quantified as a percentage compared to the control and dose response curves were developed.

### 4.4. Oil-Red O Staining and Glycerol Release Assay

Fully differentiated cells were fixed with 10% fresh formaldehyde in PBS for 1 h at room temperature and stained with filtered Oil-Red O solution (60% isopropanol and 40% water) for at least 1 h. After incubation, the wells were emptied of Oil-Red O staining solution, washed with distilled water and air dried. Images of lipid droplets in 3T3-L1 adipocytes were collected by an Olympus microscope (Tokyo, Japan). Finally, dye retained in the cells was eluted with isopropanol and quantified by measuring the optical absorbance at 500 nm using a microplate reader (Tecan Qustria GmbH, Grödig, Austria).

The glycerol levels were determined using the enzymatic reagent, free glycerol reagent (Sigma, St. Louis, MO, USA), directed by the protocol of GPO-TRINDER (Sigma, St. Louis, MO, USA).

### 4.5. Cellular ALP Activity

Cellular ALP activity of *S.* *herbacea*-treated and control cells was measured following incubation of 14 days. The cell monolayer was gently washed twice with PBS and lysed using 0.1% Triton X-100 and 25 mM carbonate buffer. The lysates were centrifuged at 4 °C 12,000× *g* for 15 min. Enzyme assay buffer (15 mM ρ-nitrophenyl phosphate, 1.5 mM MgCl_2_ and 200 mM carbonate buffer) was used to measure the ALP activity of the supernatants. The absorbance of reactive solution was measured at 405 nm.

### 4.6. RNA Extraction and Reverse Transcription-Polymerase Chain Reaction Analysis

Total RNA was isolated from 3T3-L1 and D1 adipocytes and MC3T3-E1 osteoblasts in the presence/absence of *S. herbacea* using Trizol reagent (Invitrogen Co., Carlsbad, CA, USA). For synthesis of cDNA, RNA (2 μg) was added to RNase-free water and oligo (dT), denaturated at 70 °C for 5 min and cooled immediately. RNA was reverse transcribed in a master mix containing 1× RT buffer, 1 mM dNTPs, 500 ng oligo (dT), 140 U M-MLV reserve transcriptase and 40 U RNase inhibitor at 42 °C for 60 min and at 72 °C for 5 min using an automatic T100 Thermo Cycler (Bio-Rad, Hertfordshire, UK). The target cDNA was amplified using the following sense and antisense primers: forward 5′-TTT-TCA-AGG-GTG-CCA-GTT-TC-3′ and reverse 5′-AAT-CCT-TGG-CCC-TCT-GAG-AT-3′ for PPARγ; forward 5′-TGT-TGG-CAT-CCT-GCT-ATC-TG-3′ and reverse 5′-AGG-GAA-AGC-TTT-GGG-GTC-TA-3′ for SREBP1c; forward 5′-TTA-CAA-CAG-GCC-AGG-TTT-CC-3′ and reverse 5′-GGC-TGG-CGA-CAT-ACA-GTA-CA-3′ for C/EBPα; forward 5′-CCA-CAG-CTG-AGA-GGG-AAA-TC-3′ and reverse 5′-AAG-GAA-GGC-TGG-AAA-AGA-GC-3′ for β-actin. The amplification cycles were carried out at 95 °C for 45 s, 60 °C for 1 min and 72 °C for 45 s. Final PCR products were separated by electrophoresis on 1.5% agarose gel for 30 min at 100 V after 30 cycles. Gels were then stained with 1 mg/mL ethidium bromide visualized by UV light using Davinch-Chemi imager™ (CAS-400SM, Wako Co., Osaka, Japan).

### 4.7. Real-Time RT-PCR Analysis of mRNA Expression

Gene expression was measured by real time RT-PCR in a Thermal Cycler Dice^®^ Real Time System TP800 (Takara Bio Inc., Ohtsu, Japan) following the manufacturer’s protocol. Briefly 1.0 μL of DNA sample and 12.5 μL of Maxima^®^ SYBR Green qPCR Master Mix (Fermentas, Waltham, MA, USA) containing Taq DNA polymerase, dNTP and reaction buffer were mixed. The target cDNA was amplified using the following sense and antisense primers: forward 5′-CCA-GCA-GGT-TTC-TCT-CTT-GG-3′ and reverse 5′-CTG-GGA-GTC-TCA-TCC-TGA-GC-3′ for ALP; forward 5′-GGA-CCC-GCT-GTC-TTC-TAG-TG-3′ and reverse 5′-GCC-TGC-GGT-ACA-GAT-CTA-GC-3′ for BMP-2; forward 5′-GCT-GTG-TTG-GAA-ACG-GAG-TT-3′ and reverse 5′-CAT-GTG-GGT-TCT-GAC-TGG-TG-3′ for Osteocalcin; forward 5′-GAG-CGG-AGA-GTA-CTG-GAT-CG-3′ and reverse 5′-TAC-TCG-AAC-GGG-AAT-CCA-TC-3′ for Collagen I; forward 5′-CCA-CAG-CTG-AGA-GGG-AAA-TC-3′ and reverse 5′-AAG-GAA-GGC-TGG-AAA-AGA-GC-3′ for β-actin. The PCR amplification was carried out for an initial denaturation at 95 °C for 10 min, followed by 40 PCR cycles. Each cycle proceeded at 95 °C for 15 s, 60 °C for 60 s. Relative quantification was calculated using the 2-(ΔΔCT) method. β-Actin was used as an internal control.

### 4.8. Western Blot Analysis

Western blotting was performed according to standard procedures. Briefly, cells were lysed in RIPA lysis buffer (Sigma-Aldrich Corp., St. Louis, MO, USA) at 4 °C for 30 min. Cell lysates (35 μg) were separated by 12% SDS-polyacrylamide gel electrophoresis, transferred onto a polyvinylidene fluoride membrane (Amersham Pharmacia Biotech., Amersham, England, UK), blocked with 5% skimmed milk and hybridized with primary antibodies (diluted 1:1000) against ALP and collagen I. After incubation with horseradish-peroxidase-conjugated secondary antibody at room temperature, immunoreactive proteins were detected using a chemiluminescece ECL assay kit (Amersham Pharmacia Biosciences, England, UK) according to the manufacturer's instructions. Western blot bands were visualized using a Davinch-Chemi imager™ (CAS-400SM, Wako Co., Osaka, Japan).

### 4.9. Statistical Analysis

The data were presented as mean ± SD. Differences between the means of the individual groups were analyzed using the analysis of variance (ANOVA) procedure of Statistical Analysis System, SAS v9.1 (SAS Institute, Cary, NC, USA) with Duncan’s multiple range tests. The significance of differences was defined at the *p* < 0.05 level.

## 5. Conclusions

In this study, *Salicornia herbacea* was extracted and experimented with, in order to evaluate its ability as a potential bioactive agent against osteoporosis. Results clearly indicated that, *S. herbacea* was able to inhibit adipogenesis of both pre-adipocytes and bone marrow mesenchymal cells. Inhibiting adipogenesis is important in relieving the fragile bone mass of osteoporosis which is caused by elevated adipogenesis of mesenchymal cells. In addition, results also indicated that *S. herbacea* can enhance osteoblastogenesis which is important to affect the change of fragile bone towards strong bone formation. Finally, following a bioactivity-directed isolation, two flavonoid glycosides were suggested to be responsible for the anti-osteoporosis effect of *S. herbacea* but have yet to be evaluated for their exact mechanism of action. Nonetheless, *S. herbacea* is a promising lead source for bioactive substances that can be utilized against osteoporosis.
